# Headspace Extraction of Chlorobenzenes from Water Using Electrospun Nanofibers Fabricated with Calix[4]arene-Doped Polyurethane–Polysulfone

**DOI:** 10.3390/polym14183760

**Published:** 2022-09-08

**Authors:** Hamid Najarzadekan, Muhammad Afzal Kamboh, Hassan Sereshti, Irfan Ahmad, Nanthini Sridewi, Syed Shahabuddin, Hamid Rashidi Nodeh

**Affiliations:** 1 School of Chemistry, College of Science, University of Tehran, Tehran 1417614411, Iran; 2 Department of Chemistry, Shaheed Benazir Bhutto University, Shaheed Benazirabad, Sindh 67450, Pakistan; 3 Department of Clinical Laboratory Sciences, College of Applied Medical Sciences, King Khalid University, Abha 61421, Saudi Arabia; 4 Department of Maritime Science and Technology, Faculty of Defence Science and Technology, National Defence University of Malaysia, Kuala Lumpur 57000, Malaysia; 5 Department of Chemistry, School of Technology, Pandit Deendayal Energy University, Raisan, Gandhinagar 382426, India; 6 Food Technology and Agricultural Products Research Center, Standard Research Institute, Karaj 3174734563, Iran

**Keywords:** electrospun-nanofibers, Calix[4]arene, polyurethane, polysulfone, extraction, chlorobenzenes

## Abstract

Chlorobenzenes (CBs) are persistent and potentially have a carcinogenic effect on mammals. Thus, the determination of CBs is essential for human health. Hence, in this study, novel polyurethane–polysulfone/calix[4]arene (PU-PSU/calix[4]arene) nanofibers were synthesized using an electrospinning approach over in-situ coating on a stainless-steel wire. The nanosorbent was comprehensively characterized using scanning electron microscopy (SEM) and Fourier transform infrared spectroscopy (FT-IR) techniques. The SEM analysis depicted the nanofiber’s unique morphology and size distribution in the range of 50–200 nm. To determine the levels of 1,2,4-trichlorobenzene, 1,2,3-trichlorobenzene, and 1,2,3,4-tetrachlorobenzene in water samples, freshly prepared nanosorbent was employed using headspace-solid phase microextraction (HS-SPME) in combination with gas chromatography micro electron capture detector (GC-µECD). Other calixarenes, such as sulfonated calix[4]arene, p-tert-calixarene, and calix[6]arene were also examined, and among the fabricated sorbents, the PU–PSU/calix[4]arene showed the highest efficiency. The key variables of the procedure, including ionic strength, extraction temperature, extraction duration, and desorption conditions were examined. Under optimal conditions, the LOD (0.1–1.0 pg mL^−1^), the LDR (0.4–1000 pg mL^−1^), and the R^2^ > 0.990 were determined. Additionally, the repeatability from fiber to fiber and the intra-day and inter-day reproducibility were determined to be 1.4–6.0, 4.7–10.1, and 0.9–9.7%, respectively. The nanofiber adsorption capacity was found to be 670–720 pg/g for CBs at an initial concentration of 400 pg mL^−1^. A satisfactory recovery of 80–106% was attained when the suggested method’s application for detecting chlorobenzenes (CBs) in tap water, river water, sewage water, and industrial water was assessed.

## 1. Introduction

Over the past few years, chlorinated volatile organic compounds have attracted substantial attention because they can cause environmental water contamination, which seriously affects the environment and health of human beings [1]. The chlorobenzenes (CBs) are renowned persistent environmental pollutants that widely exist in the discharged industrial effluent of various industrial units, such as the petrochemical, pharmaceutical, textile, and painting industries [1,2]. It has been determined that it is detrimental to human health for individuals to be exposed to effluents that include CBs, which can potentially produce histopathological changes, genotoxicity, mutagenicity, and carcinogenicity in humans [3]. CBs contamination has recently been identified as one of the worst health problems by the United States Environmental Protection Agency (US-EPA) and the World Health Organization (WHO) [4,5]. The maximum level of contamination goals (MLCG) was determined to be less than 0.1 μg mL^−1^. Consequently, introducing an effective analytical technique for precisely monitoring CBs in aqueous media is of prime importance and the most challenging issue of the modern era.

A review of the relevant literature revealed the importance of sample preparation techniques, such as the extraction and preconcentration of CBs, for their subsequent determination, alongside advances in modern analytical tools. For extraction, scientists have turned to many different methods, including liquid–liquid extraction, solid-phase extraction, liquid-phase microextraction, dispersive liquid–liquid microextraction, vortex-assisted liquid–liquid microextraction, single drop microextraction, and magnetic headspace adsorptive extraction. Solid-phase microextraction (SPME) [6,7] is one of these techniques, and it is both a powerful sample preparation approach and a non-exhaustive extraction method, without the need for solvents [8]. When this method is combined with GC, the fiber coating is first exposed to the sample matrix through either direct immersion or headspace mode. Next, the target analyte or analytes are thermally desorbed in the GC injection port, and are simultaneously released into the column. The commercially available sorbents for extraction of various CBs are usually made up of polymers, including polydimethylsiloxane (PDMS) and polyacrylate (P.A.), and they are coated on fused silica [9]. The conventional fibers are easily broken and are relatively expensive. Consequently, the fabrication of innovative fiber-based sorbents enhances their high capacity, imparting diverse functionalities, polarities, and stability. Additionally, wire metals have been employed in place of brittle fused silica [10].

In alkaline conditions, formaldehyde and para-substituted phenols can be cyclically condensed to generate calixarenes, which are macrocyclic ligands. The calixarene framework as a host–guest platform provides space for accepting various analytes [11]. They provide well-defined cavities, which simultaneously supply non-polar and polar features with modification of the upper-rim and lower-rim, respectively. Because of the cyclic structure of different cavity sizes and functionalities of calixarenes, they are considered suitable carriers for cationic, anionic, and nonionic species [12].

A suitable platform is required to separate, extract, and enrich target analytes. Nanofibers and nanostructured materials consist of all these properties owing to their high surface-to-volume ratio and many active sites for adsorption [13]. Recently, electrospun nanofibers have been utilized as adsorbents for SPE, micro-SPE, microextraction in the packed syringe, membrane extraction, filtration/removal, and SPME [14]. Calixarenes-electrospun nanofibers have been successfully employed for catalytic activity [15], studying toxic anion binding [16] and binding efficiency towards chromium and uranium ions [17] with polyacrylonitrile nanofibers.

This research was conducted to fabricate a novel electrospun PU–PSU/C4A nanofiber to be used as a coating for HS-SPME to extract CB compounds from water samples. Accordingly, various calix[4]arene derivatives were electrospun on the surface of a stainless steel wire and examined for headspace preconcentration of CBs under the best experimental conditions. The presence of functionalized calixarenes in the structure of nanofiber provides an appropriate force interactive with CBs, including electrostatic and π-π interactions. To our knowledge, PU–PSU/calix[4]arene has never been synthesized and used for extracting the chosen CBs as model compounds from water media.

## 2. Experimental Section

### 2.1. Chemicals and Reagents

Polyurethane (PU) and polysulfone (PSU) were purchased from Bayer Company (Leverkusen, Germany). The Merck Company (Darmstadt, Germany) provided the methanol, 1,2,4-trichlorobenzene (1,2,4-TCB), 1,2,3-trichlorobenzene (1,2,3-TCB), 1,2,3,4-tetrachlorobenzene (1,2,3,4-TCB), and N, N-dimethylformamide (DMF). The standard stock solutions of CBs were produced in methanol at a concentration of 2000 mg L^−1^ and stored at a temperature of 4 °C until their subsequent usage. Stock standard solutions of CBs were prepared, and working standard solutions were prepared daily before the extraction process.

### 2.2. Instrumentation

A gas chromatograph manufactured by Agilent (6890N, Santa Clara, CA, USA) was utilized to determine the composition of the extracted analytes. This particular model was equipped with a µ-ECD detector, an HP-5 fused silica capillary column (30 m × 0.32 mm × 0.25 µm), and a split/splitless injection port. Helium with a purity of 99.999% and a flow rate of 1 mL min^−1^, and nitrogen with a purity of 99.999% and a flow rate of 30 mL min^−1^ were employed as a carrier gas and makeup gas, respectively. 3 min of splitless injection were used to inject the sample. Temperature settings for the injector and detector were 170 °C and 290 °C, respectively. Initially, the oven was set to preheat to 70 °C for 2 min, then heated to 170 °C at a rate of 40 °C per minute for 2 min, and finally heated to 200 °C at a rate of 20 °C per minute for 5 min.

Electrospinning was carried out using a spinal needle attached to a rotating motor (to act as a collector), a syringe pump, and a direct current high voltage power source. The SPME syringe consisted of two spinal needles, an internal needle, G27 as an SPME coated needle, an external needle, and G22 as an SPME barrel.

Micrographs of nanofibers were recorded using a Zeiss DSM-960 scanning electron microscope (SEM) (Oberkochen, Germany). The functional groups of produced nanosorbent were monitored using Fourier transform infrared spectroscopy, with an Equinox 55 FTIR–A.T.R. spectrometer (Bruker, Bremen, Germany) in the 400–4000 cm^−1^ range.

### 2.3. Electrospinning

**Figure 1:**
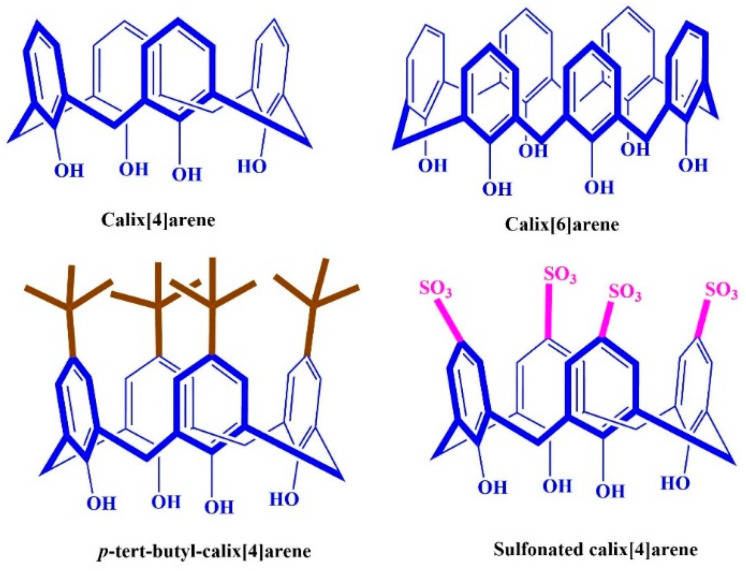
Chemical structure of calixarene derivatives.

The four calixarene derivatives (Figure 1), including calix[4]arene, sulfonated calix[4]arene, p-tert-calixarene, and calix[6]arene were synthesized according to the literature [18]. After 90 min stirring 210 mg of PU, 60 mg of PSU, and 4 mg of calixarenes in 2 mL of DMF, the mixture was sufficiently homogeneous for electrospinning.

After that, the solution was drawn into a syringe with a capacity of 2 mL and mounted on the syringe pump set. At a distance of 10 cm from the tip of the syringe needle, electrospun nanofibers were collected for 8 min. The flow rate of 0.15 mL h^−1^ and the electrospinning voltage of 15.5 kV was used. The SPME fiber was then placed in the GC inlet and heated to 150 °C for 1 h as the final step in the procedure.

### 2.4. The Procedure

At first, the PU–PSU/calix[4]arene was placed in the GC inlet and heated to 150 °C for 1 h. After that, a vial with a capacity of 10 mL was filled with 5 mL of the sample solution, which contained 100 ng mL^−1^ of each CP, and 1 g of sodium chloride. The mixture was stirred for 5 min. Afterward, the solution was sealed using a polytetrafluoroethylene septum and an open-top aluminum cap. Next, the sample solution’s headspace was allowed to interact with the fiber for a total of 5.5 min. At the end of the process, the fiber was removed, then placed into the inlet of the GC to undergo thermal desorption of the adsorbed analytes at a temperature of 170 °C for 3 min.

## 3. Results and Discussion

### 3.1. Effect of Calixarene Type

**Figure 2:**
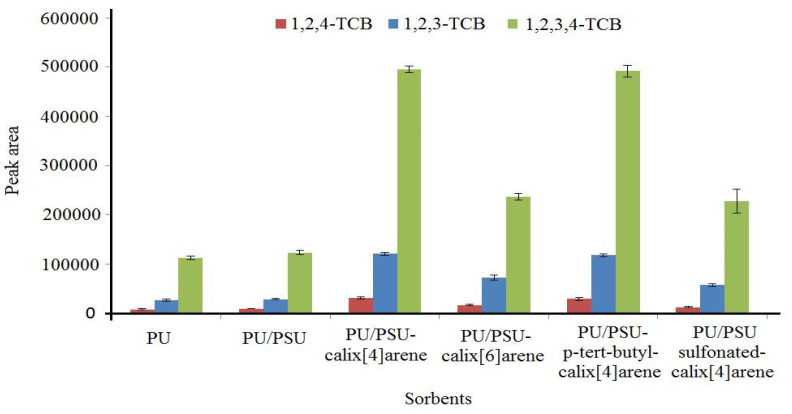
The extraction efficiency of different fabricated nanofibers.

PU–PSU nanofibers were treated with calix[4]arene, calix[6]arene, sulfonated calix[4]arene, and p-tert-butyl-calix[4]arene to see how each type of calixarene affected the extraction efficiency of the fiber. The process described in Section 2.4 was used to test the manufactured nanofibers, and the results were compared to those obtained using electrospun PU and PU–PSU nanofibers. As shown in Figure 2, utilizing nanofibers that included calixarenes improved the extraction efficiency. However, the maximum efficiency was obtained with PU–PSU/calix[4]arene nanofibers. Thus, it was selected as the adsorbent for subsequent studies.

### 3.2. Characterization

**Figure 3:**
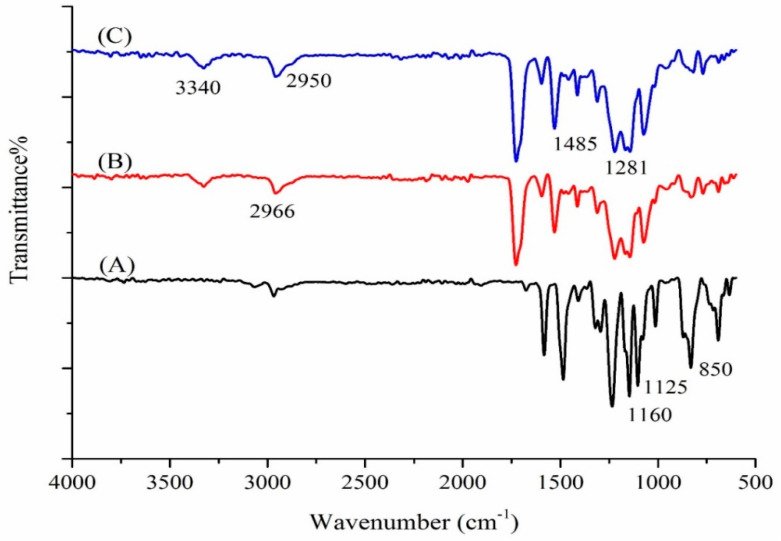
The FT-IR spectra of PSU (**A**), PU–PSU (**B**), and calix[4]arene modified PU–PSU nanofiber (**C**).

Figure 3 shows the FT-IR spectra of the PSU, PU–PSU, and PU–PSU/calix[4]arene nanofibers. The prominent peaks at 2955 cm^−1^ (C-H), 1580 and 1500 cm^−1^ (aromatic ring), 1160 cm^−1^ (S=O of sulfone), 1250 and 1090 cm^−1^ (C-O), and 850 cm^−1^ (C-S) in Figure 3A can be ascribed to the PSU [19]. Changes that correlate to PU could be seen in the spectrum of PU–PSU (Figure 3B), namely at 3391 cm^−1^ (O-H and N-H), 2955 cm^−1^ (C-H), 1726 cm^−1^ (C=O), 1582 and 1563 cm^−1^ (C-N stretching and N-H bending), 1125 cm^−1^ (C-O), and 1068 cm^−1^ (C-N) [20]. The major calix[4]arene moiety peaks (Figure 3C) have been attributed to the stretching vibrations of the O-H, C-H, C=C, and C-O functional groups, respectively, at 3340, 2950/2966, 1485, and 1281 cm^−1^ [21]. Some of these peaks overlapped with strong vibration bands of PU–PSU.

**Figure 4:**
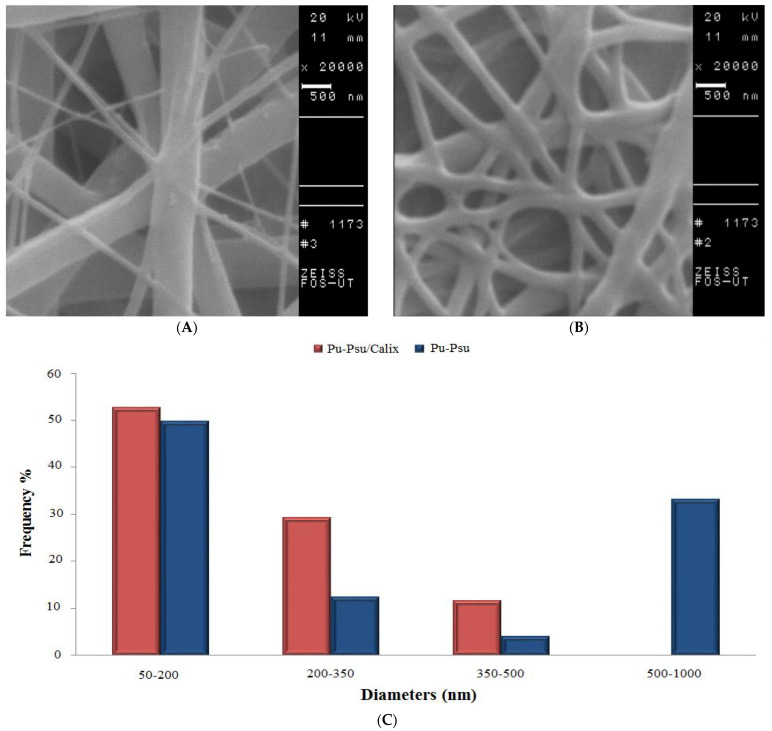
SEM micrograph of (**A**) PU–PSU nanofiber, (**B**) calix[4]arene modified PU–PSU nanofiber, and (**C**) fibers diameter histogram.

Figure 4 shows the SEM micrographs and diameter histograms of PU–PSU and PU–PSU/calix[4]arene nanofibers. The nanofibers are composed of fibers that are randomly aligned and have a smooth surface form, and they have a consistent three-dimensional porosity structure (Figure 4A,B).The fiber diameter distribution (Figure 4C) also indicates almost uniform diameters (50–450 nm) for PU–PSU/calix[4]arene as compared to PU–PSU (50–1000 nm). This is most likely because of the characteristics of the polymer solution, such as its viscosity and the effect of the high voltage electric field on the creation of nanofibers [22].

### 3.3. Impact of Ionic Strength

**Figure 5:**
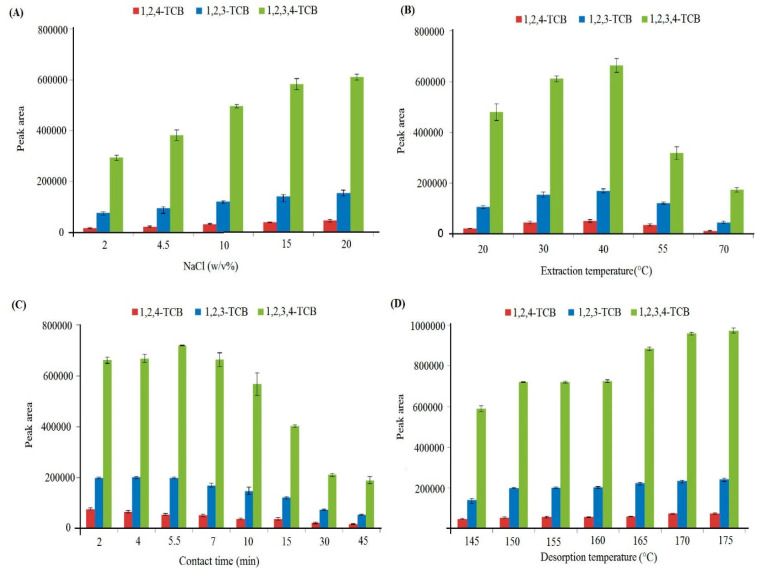
Influence of NaCl concentration (**A**), extraction temperature (**B**), extraction time (**C**), and desorption temperature (**D**) on the HS-SPME performance.

The effectiveness of the extraction was correlated with the sample solution’s ionic strength [23]. By adding 0–20% (*w*/*v*) salt (NaCl) to the sample solution, it was possible to determine how this parameter affected the extraction efficiency. As seen in Figure 5A, increasing NaCl caused the extraction efficiency to rise steadily. The analytes may migrate to the sample headspace and be absorbed by the fiber due to the salting-out phenomenon [6]. Salt concentrations above 20% (*w*/*v*) need more time for solving and preparation. For this reason, a 20% (*w*/*v*) concentration of NaCl was decided upon as the optimum condition in the extraction process.

### 3.4. Impact of Extraction Temperature

The sample solution’s temperature is a critical factor in the HS-SPME method since it impacts the extraction rate and equilibrium [24]. The analytes must be equilibrated between the headspace and both the sample solution and fiber. The increase in temperature accelerates the transfer of analytes between the solution and the headspace; thus, the partition coefficient of the headspace and sample solution (Khs/s) increases while the partition coefficient of the headspace and fiber (Khs/f) decreases [23]. The temperature range of 20–70 °C was studied to determine how temperature affected the technique’s effectiveness. As seen in Figure 5B, the efficiency improves as the temperature rises to 40 °C, then drops. Therefore, 40 °C was chosen as the best extraction temperature for subsequent tests.

### 3.5. Impact of Extraction Time

The time required for extraction is also a significant factor that affects extraction yield [25]. The reaction duration was studied by varying the contact time between the adsorbent and the sample solution from 2–45 min. It can be shown in Figure 5C that the analytical signal rose to 5.5 min, then decreased. This reduction of extraction duration after equilibrium may be due to the stainless steel wire, which gets warmer with passing time, and this phenomenon can desorb analytes of nanofiber sorbent. The rapid equilibrium period may result from the adsorbent’s high porosity and large surface area. As a result, the ideal amount of time for the extraction was determined to be 5.5 min.

### 3.6. Impact of Desorption Time and Temperature

The complete migration of analytes to the column was ensured by employing the optimum time and temperature conditions and carrying out the desorption process in the split mode. Figure 5D displays the findings from a study on desorption temperature in the split mode between 145 and 175 °C. As can be seen, raising the temperature to 170 °C improved the extraction efficiency. As can also be seen, the incorporation of PSU and calixarene into nanofibes increases the sorbent’s thermal stability. At a temperature of 170 °C, the desorption time (1–4 min) was also examined. It was determined that a desorption time of 3 min was optimal to maximize fiber lifetime, minimize the risk of coating damage, and ensure that there was no carryover impact detected.

### 3.7. Method Validation

Matrix match calibration was used to quantitatively evaluate the nanofiber’s performance under optimum conditions (salt concentration of 20% (*w*/*v*), extraction temperature of 40 °C, extraction time of 5.5 min, desorption temperature of 170 °C, and desorption time of 3 min). The limit of quantification (LOQ) was determined based on a calculation using 10 Sd/m (where Sd is the standard deviation of the blank and m represents the slope of the calibration graph), which is equal to 0.4–4 pg mL^−1^. With an acceptable R^2^ of more than 0.991, the linear dynamic range (LDR) was determined to be in the region of 0.4–1000 pg mL^−1^. The limit of detection (LOD) based on 3Sd/m was in the range of 0.1–1 pg mL^−1^. The relative standard deviation (RSD%) values (40 and 400 pg mL^−1^, *n* = 3) were in the range of 0.9–6.0%. The within-day precision was calculated on 3 different days with 3 replicates each day equal to 0.9–9.7% (C = 40 and 400 pg mL^−1^). The fiber manufactured can be utilized at least 60 times, which was determined through the sequential analysis of distilled water samples spiked with standard CB solutions at a concentration of 40 pg mL^−1^ (Table 1).

### 3.8. Analysis of Real Samples

The applicability of the developed SPME method was evaluated for the determination of the selected CBs in real samples such as tap water, sewage water (collected from the university campus), an industrial water sample (collected from an industrial park), and a river water sample (collected from Sepahsalar, Chalous, north of Iran). All the sample solutions (non-spiked and spiked) were tested in accordance with the proposed procedure (Section 2.4. The relative recoveries (RR%) were calculated using Equation (1) [6] as follows:

(1) $$\mathrm{RR}\;\left(\%\right)=\frac{C_{found}-C_{real}}{C_{added}}\times 100$$

(2) $$qe\;\left(\%\right)=\frac{V\times \left(C_{real}-C_{equal}\right)}{W}$$

where *C_found_*, *C_real_*, and *C_added_* represent the concentrations of the analytes in the real water samples that have been spiked, the concentrations of analytes in the real sample, and the concentration of the standard solution that has been added to the water samples, respectively; *qe* is the adsorption capacity and *C_equal_* represents the residual concentrations of analysts in solution after the adsorption process. In addition, *V* is the sample volume 10 mL and *W* is the fiber mass 5 mg. The results for the non-spiked samples showed no detection of the selected CBs. Table 2 displays the RR% values for the spiked samples with the target analytes’ 40 and 400 pg mL^−1^ standard solutions. The adsorption capacity of the nanofiber is calculated based on 400 pg mL^−1^ with Equation (2) [26], which is 720 pg/g.

A review of the relevant literature was carried out to evaluate the current method in comparison to other methods for calculating CBs that have been reported. Table 3 presents the findings of this evaluation. As is shown, the method used in this study has several advantages over previous studies in this area, including (i) a significantly shorter extraction time, (ii) a lower LOD, and (iii) a higher linear dynamic range (LDR).

## 4. Conclusions

In this study, the PU–PSU/calix[4]arene was fabricated and used as an effective SPME fiber coating in the headspace extraction of CBs in aquatic samples. The large surface area and porous structure of the nanofibrous PU–PSU/calix[4]arene mat provided fast adsorption of the analytes (5.5 min). The π-π stacking interactions are possibly the main reason for adsorbing CB molecules that diffused to the nanofibers/calixarene mat. The method is eco-friendly since it requires no organic solvents in the extraction and analysis steps. Moreover, the low LOD (0.1–1 pg mL^−1^) and short extraction and analysis times are characteristics that provide an excellent solution for the detection of the analytes.

## Figures and Tables

**Table 1:** Analytical figures of merit obtained for the selected CB mixtures using the HS-SPME method based on PU–PSU/calix[4]arene coupled with GC-ECD.

Compound	LOD ^a^	LOQ ^b^		LDR ^c^	R^2 d^	
1,2,4-TCB	1.0	4.0		4–800	0.9909	
1,2,3-TCB	0.1	0.4		0.4–1000	0.9911	
1,2,3,4-TCB	0.1	0.4		0.4–1000	0.9917	
	RSD% ^e^	RSD% ^f^	RSD% ^g^	RSD% ^h^	RSD% ^i^	RSD% ^j^
1,2,4-TCB	1.7	10.1	9.7	3.6	8.8	3.4
1,2,3-TCB	6.0	8.9	6.7	0.9	4.7	3.6
1,2,3,4-TCB	3.8	5.5	3.5	1.4	5.2	0.9

^a^ Limit of detection (S/N = 3, pg mL^−1^); ^b^ Limit of quantification (S/N = 10, pg mL^−1^); ^c^ Linear dynamic range (pg mL^−1^); ^d^ Determination coefficient; ^e^ Inter-day (40 pg mL^−1^); ^f^ Intra-day (40 pg mL^−1^); ^g^ Fiber to fiber (400 pg mL^−1^); ^h^ Intra-day (400 pg mL^−1^); ^i^ Inter-day (400 pg mL^−1^); ^j^ Fiber to fiber (400 pg mL^−1^).

**Table 2:** Results of HS-SPME analysis of CB mixtures using the PU–PSU/Calix[4]arene in different real water samples.

Compound	RR ^a^% (RSD%) ^b^			
40 pg mL^−1^	Industrial Water ^c^	Sewage Water ^d^	Tap Water ^d^	River Water ^e^
1,2,4-TCB	105 (6.1)	89 (5.6)	101 (3.2)	91 (7.5)
1,2,3-TCB	101 (2.8)	94 (3.3)	106 (3.9)	95 (2.4)
1,2,3,4-TCB	90 (3.4)	95 (2.3)	104 (2.3)	103 (1.7)
400 pg mL^−1^				
1,2,4-TCB	102 (3.9)	82 (5.1)	82 (3.7)	81 (2.6)
1,2,3-TCB	89 (1.8)	89 (2.8)	89 (2.2)	97 (2.0)
1,2,3,4-TCB	95 (1.0)	80 (1.8)	80 (1.8)	100 (2.5)

^a^ Relative recovery; ^b^ Relative standard deviation (*n* = 3); ^c^ Collected from an industrial park near Tehran (Iran); ^d^ Collected from our university campus; ^e^ Collected from a river in the north of Iran (Chalous city, Mazandaran province).

**Table 3:** Comparison of HS-SPME/GC-ECD analysis with other methods for determination of CBs.

Sorbent	Method	Sample	LOD (pg mL^−1^)			LDR (pg mL^−1^)	Extraction Time (min)	Recovery%	RSD%	Ref.
			1,2,4-TCB	1,2,3-TCB	1,2,3,4-TCB					
PU–PSU/calix nanofibers	HS-SPME-GC-ECD	water	1	0.1	0.1	0.4–1000	5.5	80–106	4.7–10.1	This work
Polyacrylate ^a^-SiO_2_ nanofibers	HS-SPME-GC-FID	water	5	-	-	5–1000	15	94–103	4–12	[27]
PU nanofibers	HS-SPME-GC-MS	water	10	10	10	50–1000	10	94–102	3–8	[28]
PDMS ^b^	HS-SPME-GC-MS	water	4	4	3	20–2000	30	91–107	1.8–6.7	[29]
PDMS	HS-SPME-GC-MS	soil	2.35	4.48	0.92	13.3–1333	15	-	-	[30]
Diglycidyloxycalix[4]areene	HS-SPME-GC-ECD	soil	0.2	0.34	0.18	5.33–533	15	-	-	[30]
Diglycidyloxycalix[4]areene	HS-SPME-GC-ECD	soil	0.14	0.16	0.16	0.267–26.7	15	76–100	2.9–13.4	[30]
PDMS ^b^	SPME-GC-IT-MS	soil	46	30	40	-	50	-	2–15	[31]

^a^ Polyamide, ^b^ Polydimethylsiloxane.

## Data Availability

The data presented in this study are available on request from the corresponding author.
